# *O*-GlcNAcylation of SKN-1 modulates the lifespan and oxidative stress resistance in *Caenorhabditis elegans*

**DOI:** 10.1038/srep43601

**Published:** 2017-03-08

**Authors:** Hongyuan Li, Xin Liu, Dan Wang, Liangping Su, Tingting Zhao, Zhongwei Li, Cong Lin, Yu Zhang, Baiqu Huang, Jun Lu, Xiaoxue Li

**Affiliations:** 1 The Key Laboratory of Molecular Epigenetics of the Ministry of Education, Northeast Normal University, Changchun 130021, China; 2 The Institute of Genetics and Cytology, Northeast Normal University, Changchun 130024, China

## Abstract

In *C. elegans*, the transcription factor skinhead-1 (SKN-1), the ortholog of human NF-E2-related factor 2 (Nrf-2), plays important roles in oxidative stress defense and aging processes. It has been documented that the activity of SKN-1 is regulated by its phosphorylation modification. However, whether other posttranslational modifications of SKN-1 affect its function remains unclear to date. Here we report, for the first time, that SKN-1 is *O*-GlcNAcylated at Ser470 and Thr493 by *O-*GlcNActransferase OGT-1. By generating the double mutations of Ser470/Thr493 in the wild type and *skn-1(zu67*) worms, respectively, we found that disruption of *O*-GlcNAc modification on SKN-1 repressed the accumulation of SKN-1 in the intestinal nuclei, and decreased the activities of SKN-1 in modulating lifespan and oxidative stress resistance. Moreover, under oxidative stress, SKN-1 was highly *O*-GlcNAcylated, resulting in the decrease of GSK-3-mediated phosphorylation at Ser483 adjacent to the *O*-GlcNAcylated residues (Ser470 and Thr493). These data suggest that *O*-GlcNAcylation of SKN-1 is crucial for regulating lifespan and oxidative stress resistance via the crosstalk with its phosphorylation in *C. elegans*. These findings have important implications for studying the functions of *O*-GlcNAcylation on Nrf-2 in human aging-related diseases.

Oxidative stress is a major factor of influencing aging, and is involved in a number of human diseases, such as diabetes, cancer and neurodegenerative diseases^1^,^2^. In mammalian cells, the transcription factor NF-E2-related factor 2 (Nrf2) induces the expression of phase II detoxification genes, including NAD(P)H quinone oxidoreductase 1 (NQO1), heme oxygenase-1 (HO-1), thioredoxin reductase 1 (TXNRD1), the modifier subunit (GCLM) and catalytic subunit (GCLC) of glutamate-cysteine ligase, to defense the oxidative stress^3^, whereas in *C. elegans*, skinhead-1 (SKN-1), the ortholog of Nrf-2, is required for both oxidative stress resistance and anti-aging through its accumulation in the intestinal nuclei to promote the detoxification target genes^4^. It has been proven that the subcellular location and activity of SKN-1 are regulated by its phosphorylation modification via a few signaling pathways^5^,^6^,^7^. For instance, the phosphorylation of SKN-1 by p38/mitogen-activated protein kinase (MAPK) signaling is required for SKN-1 translocation into nuclei^5^. Moreover, glycogen synthase kinase-3 (GSK-3) and insulin/IGF-1-like signaling (IIS) phosphorylate SKN-1 at specific serine and threonine sites, which prevents SKN-1 from accumulating in the intestinal nuclei and suppresses the phase II detoxification gene expressions^6^,^7^. However, it remains unknown whether the function of SKN-1 is regulated by other posttranslational modifications to date.

*O*-GlcNAcylation is a conserved posttranslational modification found in all metazoans, including worms, insects, plants and human^8^. Uridine diphosphate Nacetyl glucosamine (UDP-GlcNAc), the final product of the nutrient-sensing hexosamine signaling pathway, is the donor substrate of the *O-*GlcNActransferase (OGT)^9^,^10^,^11^. In the *O*-GlcNAc cycling, OGT catalyzes the addition of *O*-linked GlcNAc molecule from UDP-GlcNAc to serine and threonine residues of the target proteins, while *O*-GlcNAcase (OGA) is responsible for the removal of the *O*-GlcNAc modification^12^,^13^. In *C. elegans*, OGT-1 and OGA-1 are the only homologs of human OGT and OGA, respectively^14^. Abnormal *O*-GlcNAc cycling has a profound impact on diabetes, cancer and age-related neurodegenerative diseases^15^,^16^,^17^. Thus far, a large number of nuclear and cytoplasmic proteins have been known to be *O*-GlcNAcylated in eukaryote cells. Over the years, accumulating evidence has revealed the important roles of *O*-GlcNAcylation in many aspects of cellular processes, including signal transduction, transcriptional regulation, protein stability, and cell cycle control^13^,^18^,^19^,^20^,^21^,^22^. As both phosphorylation and *O*-GlcNAcylation occur at serine and threonine residues, protein *O*-GlcNAcylation has an extensive crosstalk with the protein phosphorylation during these cellular processes^14^,^23^,^24^.

*C. elegans* is a classical model for studying the regulation of aging and defense against oxidative, UV and other stresses. It has been reported that the loss of function on *ogt-1* or *oga-1* altered the lifespan and UV stress susceptibility phenotypes, suggesting *O-*GlcNAcylation plays key roles on modulating *C. elegans* aging and stress resistance^19^,^25^. However, proteins that are directly modified by *O*-GlcNAcylation to regulate the lifespan and stress resistance have not been characterized so far. Evidence has shown that both SKN-1 and OGT-1 are expressed in the intestine of worms. Furthermore, previous studies have found that the functions of some mammalian transcription factors, such as FOXO proteins, are regulated by the *O*-GlcNAc modification on these proteins^26^. Therefore, whether SKN-1 is a substrate of OGT-1 and undergoes *O*-GlcNAc modification need to unveil.

In this study, we discovered that SKN-1 was directly *O*-GlcNAcylated by OGT-1 in *C. elegans*. The loss of *O*-GlcNAc modification on SKN-1 via the site-mutations inhibited the intestinal nuclear accumulation of SKN-1 and its activities in anti-aging and oxidative stress defense. Additionally, while the oxidative stress elevated the *O*-GlcNAcylation levels of SKN-1, the GSK-3-induced phosphorylation of SKN-1 at a nearby serine residue was decreased. Together, our data identified SKN-1 as a novel target of *O*-GlcNAcylation, which is involved in modulation of oxidative stress resistance and anti-aging in *C. elegans*.

## Results

### The intestinal nuclear accumulation of SKN-1 and the expression of SKN-1 target genes are regulated by OGT-1/OGA-1

**Figure 1:**
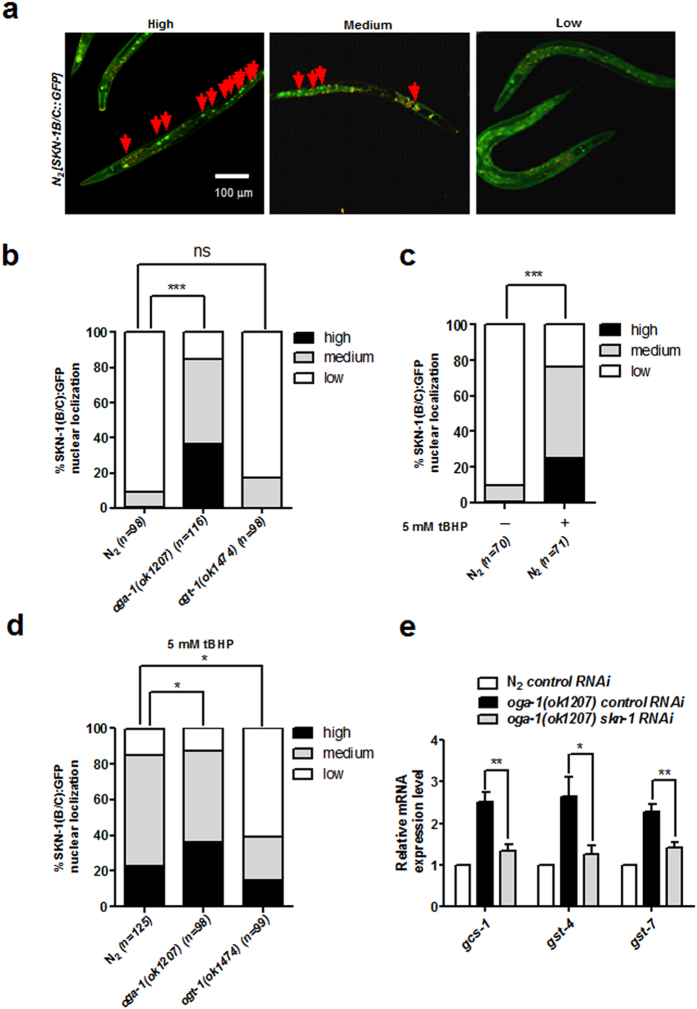
OGT-1/ OGA-1 regulated the accumulation of SKN-1 in the intestinal nuclei and SKN-1 target gene expressions. (**a**) The patterns of SKN-1B/C::GFP accumulation in the intestinal nuclei were assessed as “high”, “medium” and “low”. Arrows indicated the GFP-tagged SKN-1 proteins accumulated in the intestinal nuclei in wild-type (N_2_). (**b**) The intestinal nuclear accumulation of SKN-1B/C::GFP was promoted by loss of *oga-1*, but not *ogt-1*. (**c**) Oxidative stress increased the intestinal nuclear accumulation of SKN-1B/C::GFP in N_2_. t-butyl hydrogen peroxide (tBHP) was used as a inducer of oxidative stress. (**d**) The tBHP-induced increase of SKN-1B/C::GFP accumulation in the intestinal nuclei was up-regulated by loss of *oga-1*, but down-regulated by disruption of *ogt-1*. (**e**) Depletion of *oga-1* increased the target gene expressions of SKN-1. The mRNA levels of SKN-1 target genes were measured by RT-qPCR in wild type (N_2_) and *oga-1(ok1207*) worms treated with control dsRNA, and *oga-1(ok1207*) worms treated with *skn-1* dsRNA. *act-1* was used as an internal reference. All of the representative data were from at least three independent experiments. *p < 0.05; **p < 0.01; ***p < 0.001; ns, no significance

The accumulation of SKN-1 in the intestinal nuclei is the core step to stimulate the activities of this transcription factor in *C. elegans*. In order to know whether there is the relationship between *O*-GlcNAc cycling and the activities of SKN-1, we first analyzed the changes of SKN-1’s accumulation in the intestinal nuclei in *ogt-1* and *oga-1* mutants. The functions of *oga-1* and *ogt-1* were first proven depleted in *C. elegans*, by measuring the global *O*-GlcNAcylation level in these two mutants (Supplementary Fig. S1). The levels of SKN-1B/C::GFP accumulation in the intestinal nuclei were assigned as “high”, “medium” and “low” according to a well-acknowledged approach as described in the method^4^,^6^ (Fig. 1a). Results showed that the level of SKN-1B/C::GFP accumulation in the intestinal nuclei was increased in *oga-1(ok1207*), but not *ogt-1(ok1474*) (Fig. 1b). Since accumulating evidence has shown that the intestinal nuclear accumulation of SKN-1 can be triggered by oxidative stress^4^,^5^,^6^, we treated N_2_ with 5 mM t-butyl hydrogen peroxide (tBHP) according to the previous publications^4^,^6^,^7^. Addition of tBHP increased reactive oxygen species (ROS) level in the whole worms by 2-fold as shown by changes in 2, 7-dichlorodihydrofluorescein fluorescence (Supplementary Fig. S2). Moreover, it can be stated 5 mM tBHP treatment significantly increased the intestinal nuclear accumulation of SKN-1B/C::GFP in N_2_ (Fig. 1c), while only slightly increased in the *oga-1* mutants (Fig. 1d). However, when the *ogt-1* mutants were treated with tBHP, a lower percentage of the SKN-1B/C::GFP positive intestinal nuclei was observed, compared with N_2_ (Fig. 1d). At last, by the RT-qPCR analysis, we found the mRNA expression levels of SKN-1 target genes, such as *gcs-1, gst-4* and *gst-7*, were up-regulated in the *oga-1* mutants, which were depleted by *skn-1* RNAi (Fig. 1e and Supplementary Fig. S3). These results suggest that *O-*GlcNActransferase OGT-1 and *O*-GlcNAcase OGA-1 in *C. elegans* play important roles on regulating the accumulation of SKN-1 in the intestinal nuclei and SKN-1’s target gene expressions.

### SKN-1 interacts with and is *O-*GlcNAcylated by OGT-1

**Figure 2:**
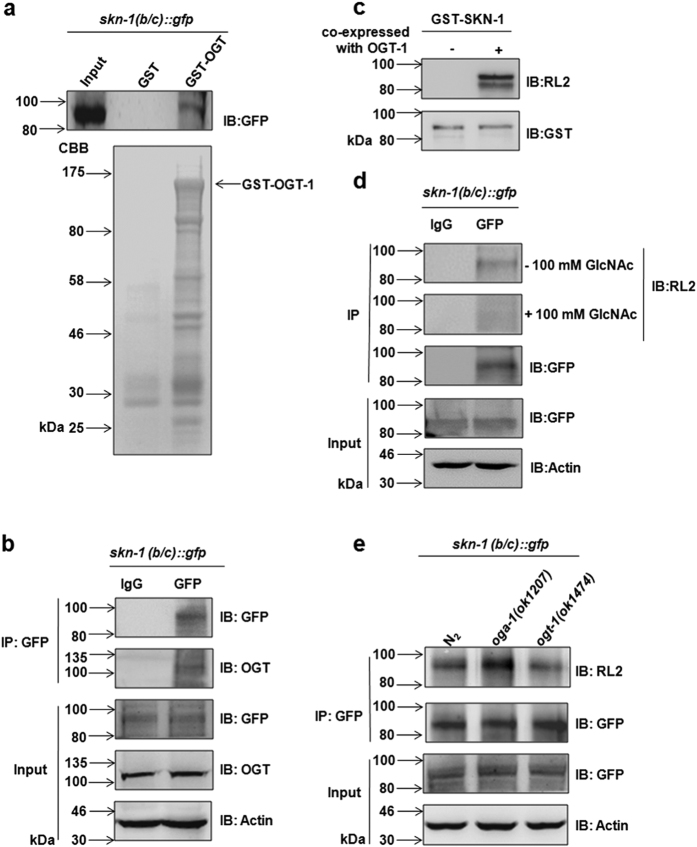
SKN-1 interacted with and was *O*-GlcNAcylated by OGT-1. (**a**) GST-OGT-1 bound to SKN-1 proteins expressed in the wild type worms. GST pull-down assay was performed by IP with GST-OGT-1 protein from the whole extracts of the wild-type (N_2_) expressing *SKN-1(B/C)::GFP*, and followed by immunoblotting with anti-GFP antibody. The arrow on the right side indicated the band of GST-OGT-1 purifed from *E. coli* BL21. GST protein was as the negative control. (**b**) OGT-1 interacted with SKN-1 *in vivo*. co-IP was operated by utilizing the whole extracts of *SKN-1B/C::GFP* worms with anti-GFP antibody, followed by immunoblotting with anti-OGT antibody. IgG was as the negative control. **(c)** SKN-1 was *O-*GlcNAcylated by OGT-1. In *O*-GlcNAcylation assay, the *O*-GlcNAcylated SKN-1 was purified and detected with the anti-*O*-GlcNAc antibody upon the co-expression of HIS-OGT-1 and GST-SKN-1 in *Escherichia coli* BL21. (**d**) SKN-1 was *O-*GlcNAcylated in *C.elegans*. SKN-1 proteins were immunoprecipitated with anti-GFP antibody from the whole extracts of *SKN-1B/C::GFP* worms. The *O*-GlcNAcylation of SKN-1was detected by immunoblotting with anti-*O*-GlcNAc antibody. 100 mM free GlcNAc was pre-incubated with anti-*O*-GlcNAc antibody during immunoblotting to confirm the specific signal of the *O*-GlcNAcylated SKN-1. IgG was as the negative control. (**e**) OGT-1*/*OGA-1 regulated the *O*-GlcNAcylation cycle of SKN-1. SKN-1 proteins were immunoprecipitated with anti-GFP antibody by using the whole extracts from the *oga-1(ok1207*), *ogt-1(ok1474*) and wild-type (N_2_) expressing *SKN-1B/C::GFP*, respectively. Then the *O*-GlcNAcylation level of SKN-1 was measured by immunoblotting with anti-*O*-GlcNAc antibody. IB means immunoblotting.

Next, we examined whether OGT-1 could interact with and *O*-GlcNAcylate SKN-1. GST pull-down assays were performed by utilizing the whole-worm extracts from N_2_ expressing *SKN-1(B/C)::GFP*. The results showed that SKN-1 was able to bind to GST-OGT-1, but not GST (Fig. 2a).To confirm their physiological interaction *in vivo*, we performed immunoprecipitation (IP) with anti-GFP antibody in *SKN-1(B/C*):*:GFP* worms and found that OGT-1 interact with SKN-1 in the worm extracts (Fig. 2b).

To further investigate whether SKN-1 could be directly *O*-GlcNAcylated by OGT-1, we performed the *O*-GlcNAcylation assay according to the method reported previously^27^. The constructs expressing HIS-tagged OGT-1 and GST-tagged SKN-1 were co-transfected into *Escherichia coli* BL21. Then, the GST-SKN-1 was purified with glutathione Sepharose 4B beads and examined by immunoblotting with anti-*O*-GlcNAc antibody (RL2). As expected, SKN-1 exhibited a strong *O*-GlcNAc modification signal (Fig. 2c). To prove whether SKN-1 could also be *O*-GlcNAcylated *in vivo*, IP was performed with anti-GFP antibody using whole extracts of *SKN-1(B/C*):*:GFP* worms. The specific signal of *O*-GlcNAcylated SKN-1 was observed in these worms, which was further confirmed via the GlcNAc competition experiment by pre-incubation of anti-*O*-GlcNAc antibody with free GlcNAc during immunoblotting (Fig. 2d).

Importantly, extracts of *oga-1* and *ogt-1* mutant worms expressing *SKN-1(B/C*)::*GFP* were then used in the IP assays. While a higher level of *O*-GlcNAc modification of SKN-1 was observed in *oga-1* mutants, a lower level was detected in *ogt-1* mutants, compared to N_2_ (Fig. 2e). Taken together, these data suggest that SKN-1 was *O*-GlcNAcylated by OGT-1 in *C. elegans.*

### SKN-1 is *O-*GlcNAcylated preferentially at Ser470 and Thr493 by OGT-1

**Figure 3:**
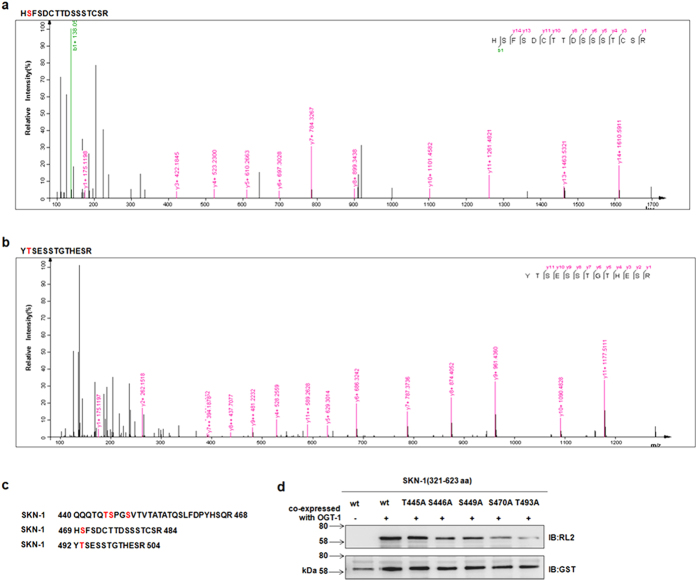
SKN-1 was *O-*GlcNAcylated at Ser470 and Thr493 primarily. (**a**,**b**) The *O*-GlcNAcylation sites of SKN-1 were analyzed by mass spectrometry. The residues of *O*-GlcNAcylation sites Ser470 and Thr493 were identified from the fragmentation of the SKN-1 peptides HSFSDCTTDSSSTCSR and YTSESSTGTHESR by mass spectrometry. (**c**) The *O*-GlcNAcylated residues were shown in the animo acid sequnce of the analyzed SKN-1 peptides. the *O*-GlcNAcylated residues of SKN-1 were indicated in red. (**d**) Ser470 and Thr493 were the major *O-*GlcNAcylation sites catalyzed by OGT-1. Each serine or threonine of the idetified *O*-GlcNAcylation sites on SKN-1(321–623aa) peptide was mutated into alanine. GST-OGT-1 and SKN-1 peptides (wild type or mutant) were co-expressed in BL21. The *O*-GlcNAcylation level were then measured by using anti-*O*-GlcNAc antibody. IB means immunoblotting.

To identify the specific serine and threonine residues of SKN-1 modified by OGT-1, we next purified GST-tagged SKN-1 from *E. coli* BL21, in which GST-tagged SKN-1 plasmids and HIS-tagged OGT-1 plasmids were co-transformed. Then, the mass spectrometric (MS) analysis was conducted according to the previous study^27^. The results revealed that the sites of Thr445, Ser446, Ser449, Ser470 and Thr493 of SKN-1 were *O*-GlcNAcylated (Fig. 3a–c and Supplementary Fig. S4a,b). Then, each of these *O*-GlcNAc modification sites was individually mutated into alanine, and co-expressed with HIS-tagged OGT-1 in BL21. By using the *O-*GlcNAcylation assay, we found that the *O*-GlcNAcylation level of SKN-1 was decreased significantly with the single point mutations of Ser470 and Thr493 (Fig. 3d). These data indicate that the Ser470 and Thr493 of SKN-1 are the major *O*-GlcNAcylation sites catalyzed by OGT-1.

### Ser470/Thr493 *O-*GlcNAcylation of SKN-1 regualtes its functions in lifespan and oxidative stress resistance

**Figure 4:**
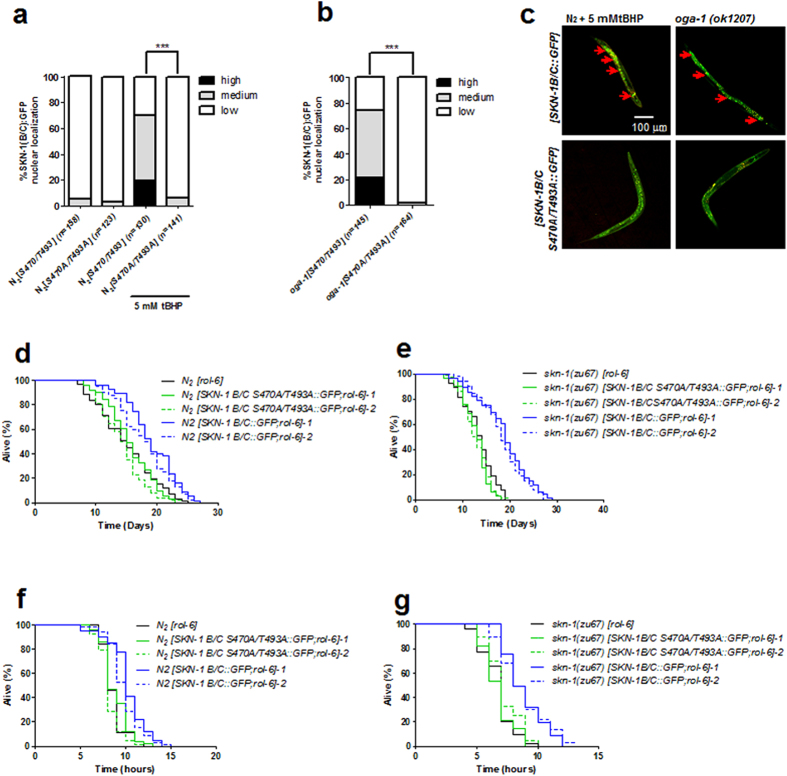
Ser470/Thr493 *O-*GlcNAcylation of SKN-1 regualted its intestinal nuclear accumulation and functions on lifespan and oxidative stress resistance. (**a**) The double mutations of Ser470/Thr493 down-regulated the intestinal nuclear accumulation of SKN-1 promoted by tBHP treatment. The intestinal nuclear accumulation of SKN-1 was scored in the wild type worms expressing *SKN-1B/C::GFP* or *SKN-1B/C S470A/T493A::GFP*, with or without tBHP treatments. (**b**) Loss of *O*-GlcNAcylation at Ser470/Thr493 inhibited the increase in the intestinal nuclear accumulation of SKN-1 induced by disruption of *oga-1*. The intestinal nuclear accumulation of SKN-1 was scored in *oga-1(ok1207*) worms expressing *SKN-1B/C::GFP* or *SKN-1B/C S470A/T493A::GFP* under normal condition. (**c**) The presentative images to show the intestinal nuclear accumulation of SKN-1 described A and B. The accumulated SKN-1 in the intestinal nuclei was observed by confocal microscopy (arrows). (**d**,**e**) The loss of Ser470/Thr493 *O*-GlcNAcylation depressed the function of SKN-1 on longevity. The lifespan was assayed in the wild type worms and *skn-1(zu67*) worms harboring *rol-6;SKN-1B/C::GFP* and *rol-6;SKN-1B/C S470A/T493A* transgenes, respectively. (**f,g**) The depletion of Ser470/Thr493 *O*-GlcNAcylation on SKN-1 resulted in its decrease of oxidative stress defense. The wild type and *skn-1(zu67*) worms expressing *rol-6;SKN-1B/C::GFP* and *rol-6;SKN-1B/C S470A/T493A* transgenes were treated with 9.125 mM tBHP in NGM plates as oxidative stress, and following the survival analysis respectively. All the representative data were from at least three independent experiments. ***p < 0.001.

To investigate whether *O*-GlcNAc modification at Ser470 and Thr493 is crucial for the functions of SKN-1 in *C. elegans*, the *SKN-1B/C S470A/T493A::GFP* transgenic worms with the loss of *O*-GlcNAcylation on SKN-1 were generated on the wild type background. Treatment with tBHP increased the intestinal nuclear accumulation of SKN-1 in *SKN-1B/C::GFP* worms, but not in *SKN-1B/C S470A/T493A::GFP* worms (Fig. 4a,c). Furthermore, the increase in the level of SKN-1 in intestinal nuclei, which was induced by loss of *oga-1*, was significantly repressed by the expression of *SKN-1B/C S470A/T493A::GFP* in *oga-1* mutants (Fig. 4b,c).

Next, we analyzed the lifespan and oxidative stress tolerance of N_2_, in which *SKN-1B/C::GFP* and *SKN-1B/C S470A/T493A::GFP* transgenes were expressed, respectively. The results showed that the overexpression of *SKN-1B/C::GFP* increased the longevity and oxidative stress tolerance of N_2_ (Fig. 4d,f and Supplementary Table S1,2). While, the lifespan and their resistance to oxidative stress of N_2_ were not altered by expressing *SKN-1B/C S470A/T493A::GFP* (Fig. 4d,f and Supplementary Table S1,2).

Furthermore, the *SKN-1B/C::GFP* and *SKN-1B/C S470A/T493A::GFP* transgenic worms were then generated on the *skn-1(zu67*) background. We found that the lifespan of *skn-1(zu67*) worms were extended by the overexpression of *SKN-1B/C::GFP*, but not *SKN-1B/C S470A/T493A::GFP* (Fig. 4e and Supplementary Table S1). In addition, analysis of oxidative stress sensitivity revealed that *SKN-1B/C S470A/T493A::GFP* did not contribute to the oxidative stress resistance in the *skn-1(zu67*) worms (Fig. 4g and Supplementary Table S2). Apparently, the *O*-GlcNAcylation of SKN-1 at Ser470/Thr493 is essential for promoting SKN-1 accumulation in the intestinal nuclei, and activating its function in modulating lifespan and oxidative stress resistance in *C. elegans*.

### Oxidative stress enhances the *O-*GlcNAcylation of SKN-1, resulting in the decrease of phosphorylation at Ser483 by GSK-3

**Figure 5:**
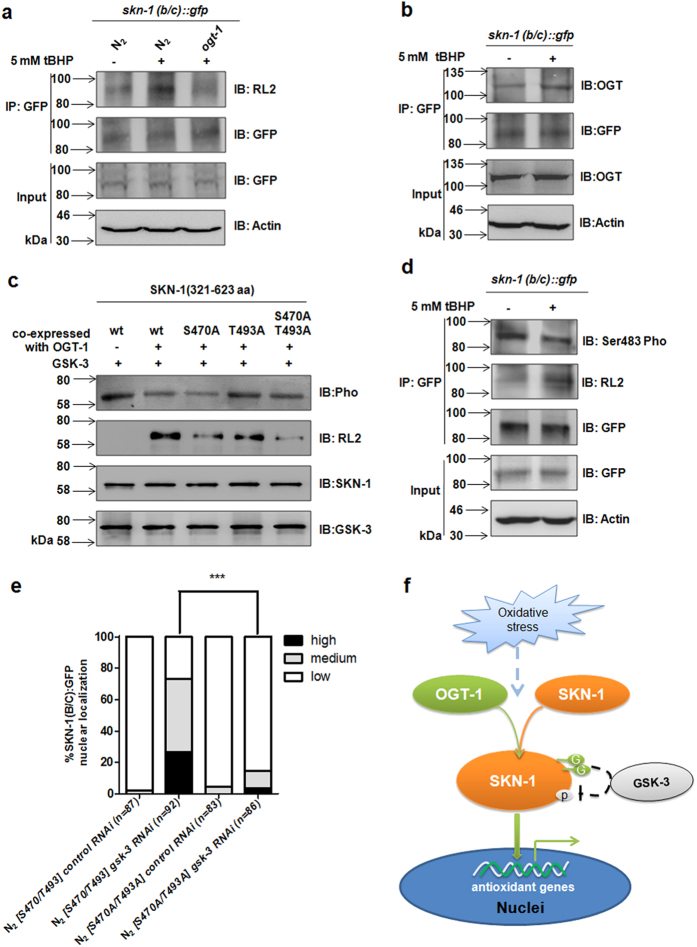
Oxidative stress increased the *O-*GlcNAcylation level of SKN-1, resulting in the decrease of GSK-3-mediated phosphorylation of Ser483. (**a**) Oxidative stress enhanced the *O*-GlcNAcylation level of SKN-1. The immunoprecipitations were performed by using anti-GFP antibody from the whole extracts of the wild type (N_2_) or *ogt-1(ok1474*) worms expressing *SKN-1B/C::GFP* with or without tBHP treatments. The *O*-GlcNAcylation level of SKN-1 was detected by immunoblotting with anti-*O*-GlcNAc antibodies. The *SKN-1B/C::GFP* worms on the wild type (N_2_) background without tBHP treatment were as the negative control. (**b**) Oxidative stress elevated the interaction between OGT-1 and SKN-1. The wild type worms expressing *SKN-1B/C::GFP* were treated with tBHP or not. SKN-1 was then immunoprecipitated from the whole cell extracts with anti-GFP antibody, followed by immunoblotting with anti-OGT antibody. (**c**) The *O*-GlcNAcylation of SKN-1 down-regulated GSK-3-mediated phosphorylation *in vitro*. Recombinant GSK-3 were incubated with wild type SKN-1(321–623aa), or the SKN-1(321–623aa) peptides with single mutation of S470A or T493A, or double mutations of S470A/T493A, respectively. The phosphorylation level and *O*-GlcNAcylation level of the wild type or mutant SKN-1 proteins were then detected by immunoblotting with the anti-phosphoserine antibody (Pho) and anti-*O*-GlcNAc antibody. (**d**) Oxidative stress decreased the phosphorylation level of SKN-1 at Ser483. SKN-1 proteins were immunoprecipitated with anti-GFP antibody by using the whole cell extracts of *SKN-1B/C::GFP* worms with or without tBHP treatment, followed by immunoblotting with anti-phospho-SKN-1(Ser483) antibody (Ser483 Pho) and anti-*O*-GlcNAc antibody. (**e**) The loss of *O-*GlcNAcylation on SKN-1 at Ser470/Thr493 inhibited its intestinal nuclear accumulation promoted by *gsk-3* RNAi. The wild type worms harboring *SKN-1B/C S470A/T493A* or *SKN-1B/C::GFP* transgenes were treated with *gsk-3* RNAi and control RNAi, respectively. The accumulation of SKN-1 in the intestinal nuclei was then scored in these worms. All of the representative data from at least three independent experiments. ***p < 0.001. (**f**) The roadmap illustrating that *O*-GlcNAcylation of SKN-1 regulates its intestinal nuclear accumulation and functions on anti-aging and the oxidative stress resistance in *C. elegans*.

Since our data showed that the tBHP-induced accumulation of SKN-1 in the intestinal nuclei was suppressed by lacing of *O*-GlcNAcylation on SKN-1, we next intended to determine whether this modification functions as the response to the oxidative stress. Therefore, the wild type worms harboring *SKN-1(B/C)::GFP* expression were treated with tBHP, and followed by co-IP experiments. The results showed that the *O*-GlcNAcylation level of SKN-1 was elevated by this oxidative stress in the wild type worms, but not in the *ogt-1* mutants (Fig. 5a). Meanwhile, tBHP treatment increased the binding of OGT-1 to SKN-1 (Fig. 5b). These data suggest *O*-GlcNAcylation of SKN-1 is a physical response in *C. elegans* to defense against the oxidative stress.

It has been documented that the crosstalk between *O*-GlcNAcylation and phosphorylation usually occurs at the residues close to each other^28^. Thus, we examined the sequence surrounding Ser470 and Thr493, and focused on Ser483, which has been reported to be phosphorylated by GSK-3^6^. (Supplementary Fig. S5). The antibody specifically recognizing the phosphorylated Ser483 of SKN-1 was prepared (Supplementary Fig. S6). By using this antibody, we found that loss of *oga-1* increased the *O*-GlcNAcylation level of SKN-1, while decreased the phosphorylation level of Ser483 (Supplementary Fig. S7). As a substantial elevation of GSK-3 in both *ogt-1* and *oga-1* mutants has been reported previously^29^, we then examined whether *O*-GlcNAcylation of SKN-1 inhibits the phosphorylation of Ser483 directly by GSK-3 kinase assay *in vitro*. The GST-SKN-1(321–623aa) co-expressed in *E. coli* BL21 with or without HIS-OGT-1, was purified and used as the substrate of GSK-3. The kinase assays revealed that the phosphorylation level of SKN-1 was down-regulated by the *O*-GlcNAcylation of SKN-1. In contrast, this phosphorylation level was restored upon the single mutation of T493A, as well as the double mutations of S470A and T493A (Fig. 5c). These data indicate that the *O*-GlcNAcylation of SKN-1 is able to suppress the GSK-3-mediated phosphorylation on this protein directly.

To further confirm the crosstalk between *O*-GlcNAcylation and GSK-3-mediatd phosphorylation on SKN-1 at Ser483 under oxidative stress in *C. elegans*, the wild-type (N_2_) expressing *SKN-1(B/C)::GFP* were treated with tBHP, followed by IP assay. As expected, the phosphorylation level of SKN-1 at Ser483 was decreased in the tBHP-treated worms, which had a higher *O*-GlcNAcylation level of SKN-1 compared with the untreated worms (Fig. 5d).

Moreover, we knocked down the *gsk-3* in *SKN-1B/C::GFP* and *SKN-1B/C S470A/T493A::GFP* transgenic worms, respectively. As expected, the intestinal nuclear accumulation of SKN-1 was significantly increased by *gsk-3* RNAi in the *SKN-1B/C::GFP* worms, but rarely raised in the *SKN-1B/C S470A/T493A::GFP* worms (Fig. 5e). These results show that the oxidative stress increases the *O*-GlcNAcylation level of SKN-1, leading to the decrease of GSK-3-mediated phosphorylation on Ser483 *in C. elegans*.

## Discussion

SKN-1, the ortholog of mammalian Nrf2, is a crucial transcription factor engaged in modulation of oxidative stress resistance and longevity, whose functional regulation is predominantly achieved by posttranscriptional modification^4^,^5^,^6^,^7^,^30^. Unlike Nrf2, of which many types of protein modification have been extensively documented, such as phosphorylation, ubiquitination, acetylation and sumoylation^31^,^32^,^33^,^34^, SKN-1 is primarily as the substrate of phosphorylation^5^,^6^,^7^,^30^. In the present study, we identified for the first time that SKN-1 is *O*-GlcNAcylated at Ser470 and Thr493 by OGT-1 in *C. elegans*. This modification of SKN-1 plays a crucial role on promoting the activities of SKN-1 in modulating lifespan and the tolerance to oxidative stress.

It is well known that the ortholog of *C. elegans* OGT-1 in human, named OGT, regulates the global levels of *O*-GlcNAc modification of cells, and has a large number of substrates^35^,^36^,^37^. To investigate whether Nrf2 could be a substrate of OGT in human cells, we performed *O*-GlcNAcylation assay and found Nrf2 was transiently *O*-GlcNAcylated by human OGT *in vitro* (Supplementary Fig. S8). Therefore, the physiological and pathological roles of Nrf2’s *O*-GlcNAcylation in human yet to be further explored.

It is noteworthy that loss of *oga-1* or *ogt-1* did not remarkably affect the SKN-1 expression in the polymodal sensory neurons (ASI neurons) (Supplementary Fig. S9). ASIs are important polymodal sensory neurons, which are regarded as the putative hypothalamus in *C. elegans*. It has been reported SKN-1 expression in these neurons is important to extend lifespan through dietary restriction (DR)^38^. This suggests that the *O*-GlcNAc modification of SKN-1 might regulate *C. elegans* lifespan independent of DR pathway. We found that loss of *ogt-1* decreased the intestinal nuclear accumulation of SKN-1 promoted by tBHP treatment. However, some percentage of SKN-1 was still observed in the intestinal nuclei in *ogt-1* mutant with tBHP treatment (Fig. 1d). Preliminary studies also reported that disruption of *ogt-1* did not completely eliminate the intestinal nuclear accumulation of SKN-1 induced by sodium azide^39^. These data imply some factors might exist in *C. elegans* to counteract the decrease in the accumulation of SKN-1 in the intestinal nuclei triggered by loss of *ogt-1*.

Mounting evidence has suggested that *O*-GlcNAc plays a critical role in regulating chromatin structure^40^. Some *O*-GlcNAc sites on histones H2A, H2B, and H4 were identified by mass spectrometry, and the changes of histone *O*-GlcNAcylation were detected during mitosis and with heat shock^41^,^42^. Our results showed that the expressions of SKN-1 target genes were up-regulated by the loss of *oga-1* (Fig. 1e). Therefore, we further investigated whether the transcriptional expressions of SKN-1 target genes can be promoted by the histone *O*-GlcNAcylation on their promoters. According to the published data of whole-genome chromatin immunoprecipitation (ChIP)-on-chip and transcriptional profiling on the wild-type, *oga-1* and *ogt-1* mutant worms^19^, few positive signals of histone *O*-GlcNAcylation were detected on the promoters of phase II detoxification genes, which were known as the classical target genes of SKN-1. These data further verify that the activities of SKN-1 on anti-aging and oxidative stress resistance are regulated by the *O*-GlcNAc modification on this transcription factor itself.

The existing evidence has pointed to the extensive crosstalk between *O*-GlcNAcylation and phosphorylation that play important roles in various aspects of cellular processes^14^,^21^,^24^,^28^. Since the sites of *O*-GlcNAcylation on SKN-1 discovered in our study are close to Ser483, which is reported phosphorylated by GSK-3, we further explored the crosstalk between GSK-3-mediated phosphorylation and *O*-GlcNAcylation of SKN-1.The GSK-3 kinase assays showed that the phosphorylation level of SKN-1 mediated by GSK-3 was directly inhibited by the *O*-GlcNAcylation of this protein at Ser470 and Thr493 (Fig. 5c). Moreover, *gsk-3* RNAi failed to elevate the intestinal nuclear accumulation of SKN-1 in the *SKN-1B/C S470A/T493A::GFP* worms, indicating that the *O*-GlcNAc modification of SKN-1 was required for maintaining the functions of SKN-1 controlled by GSK-3 pathway. Previous studies have reported the phosphorylation of SKN-1catalyzed by other kinases, such as AKT-1 and AKT-2 in the Insulin/IGF-1-like pathway, can also inhibit the accumulation of SKN-1 in the intestinal nuclei and decrease its activities in regulating lifespan and oxidative stress resistance^7^. Therefore, it is possible that there is the relationship between the *O*-GlcNAcylation of SKN-1 and its phosphorylation mediated by other kinases.

Taken together, our findings identify a new posttranslational modification *O*-GlcNAcylation of SKN-1. This modification, as a response of the oxidative stress, may directly inhibit its phosphorylation mediated by GSK-3, to stimulate the expression of its target genes involved in regulating lifespan and oxidative stress resistance in *C. elegans* (Fig. 5f). Notably, we find the *O*-GlcNAcylation level of SKN-1 was increased in response to oxidative stress, indicating that the OGT-1-mediated *O*-GlcNAcylation of SKN-1 is a dynamic process. The factors that stimulate OGT-1 to interact with and modify SKN-1, thereby activating the downstream target genes, should be unveiled further. Importantly, these data provide the evidence for the conserved function of the *O*-GlcNAcylation of Cap’n’Colla transcription factors over the evolution.

## Methods

### Strains

The following strains were used in this work: N_2_ Bristol (wild-type), *oga-1(ok1207*); *ogt-1(ok1474*), LD1(*ldls7*) were provided by the Caenorhabditis Genetic Center (CGC), *oga-1(ok1207*) and *ogt-1(ok1474*) were crossed to LD1(*ldls7*) to generate *oga-1(ok1207);ldls7, ogt-1(ok1474);ldls7* strains. The strains of *oga-1(ok1207*) and *ogt-1(ok1474*) from CGC were backcrossed at least three times before used. The transgenic worms: N_2_ [*rol-6*], N_2_ [*SKN-1B/C::GFP;rol-6*], N_2_ [*SKN-1B/C S470A/T493A::GFP;rol-6*], *skn-1(zu67*) [*rol-6*], *skn-1(zu67*)[*SKN-1B/C::GFP;rol-6*], *skn-1(zu67*)[*SKN-1B/C S470A/T493A::GFP;rol-6*], *oga-1(ok1207*) [*rol-6*], *oga-1(ok1207*) [*SKN-1B/C::GFP;rol-6*], *oga-1(ok1207*) [*SKN-1B/C S470A/T493A::GFP;rol-6*] were made in our lab.

### Transgenesis

The over expression plasmid SKN-1B/C::GFP was a generous gift from Prof. T. Keith Blackwell (Research Division, Joslin Diabetes Center). We created the mutant constructs of SKN-1B/C S470A/T493A::GFP by using the protocol of QuikChange (Stratagene). The transgenic strains were generated by injecting plasmid into the germlines of young adult animals. The constructs SKN-1B/C::GFP and SKN-1B/C S470A/T493A::GFP were respectively injected into wild-type (N_2_), *oga-1(ok1207*) and *skn-1(zu67*) at 10 ng/μl, along with the rol-6 marker (pRF4) at 50 ng/μl.

### Lifespan analysis

Prior to experiments, all animals were maintained at the permissive temperature and grown for at least two generations in the presence of food to assure health. Lifespan analyses were conducted at 20 °C. Synchronized L1 worms were fed with OP50, grown to young adult, and then 30 worms were transferred to a new plate. Animals were tapped every day and scored as dead when they did not respond to the platinum wire pick. All of the life span assays were repeated at least three times. Survival plots, *p* values (Log-Rank), and proportional hazards were determined by using GraphPad Prism 5 software.

### Oxidative stress resistance assay

To examine the accumulation of SKN-1B/C::GFP in the intestinal nuclei and SKN-1 target gene expressions by RT-qPCR under oxidative stress, young adults were exposed to 5 mM t-butyl hydrogen peroxide tBHP (Sigma) for 90 minutes. To assess the viability, day 1 later adults were transferred to NGM (nematode growth media) plates containing tBHP at 9.125 mM. Animals were incubated at 20 °C and periodically scored for survival.

### Cellular localization of SKN-1B/C::GFP

The animals were incubated for 24 h at 20 °C and allowed to lay eggs. Their surviving progeny were grown to L4 larvae and young adults, and then the accumulation of SKN-1B/C::GFP in the intestinal nuclei was scored according to the protocol in the previous study^4^,^5^. Briefly, “High” indicates that a strong SKN-1B/C::GFP signal is present in all of the intestinal nuclei. “Medium” indicates that nuclear SKN-1B/C::GFP is present at high levels anteriorly, posteriorly, or anteriorly and posteriorly, but barely detectable midway through the intestine, or that a weak SKN-1B/C::GFP signal was observed in all intestinal nuclei. “Low” indicates that SKN-1B/C::GFP is barely detectable in the intestinal nuclei.

### RNA isolation and quantitative PCR

To prepare worm sample, animals were picked and placed to clean plates to minimize contamination. Then approximately 200 animals suspended in 50 μl M9 buffer. Total RNAs from worms and cells were prepared by using Trizol reagent kit (TaKaRa) according to the manufacturer’s instructions. The cDNA was generated with oligo(dT) primers (Promega) by using the Reverse Transcription System (Promega). The Quantitative Real Time PCR was carried out using the SYBR Green Real time PCR Master Mix (TOYOBO) and operating on a Light Cycler 480 Real-Time PCR system Roche and then normalized to *act-1* for worms.

### Plasmids, reagents and antibodies

The cDNAs for *skn-1, skn-1*(321–623aa) and *ogt-1* of *C. elegans* were amplified by PCR and cloned into pGEX-6P-1 and PET-28a vectors, respectively. All of the point mutations of SKN-1 were generated by site-directed mutagenesis according to the QuikChange (Stratagene) protocal. The antibodies of anti-GFP, anti-GST (Tianjin Sungene Biotech), anti-β-actin (Sigma), anti-*O-*GlcNAc RL2 (Abcam), anti-OGT (sigma) and anti- phosphoserine (Millipore) were purchased from the commercial channels. The anti-OGT and anti-GFP antibodies were first analyzed and make sure of their specific binding to the OGT-1 and GFP in *C. elegans* (Supplementary Fig. S10a,b). Rabbit polyclonal antibodies against phospho-SKN-1(Ser483) were generated by using synthetic peptides, TTDSSSTCS(#)RLSSESPRYTSE. # means the phosphorylated residue Ser483.

### In *vitro* pull-down assays

The proteins of GST and GST-OGT-1 were expressed in BL21(DE3) bacteria and purified with glutathione Sepharose 4B beads (for GST fusion proteins, GE Healthcare), respectively. GST-fusion protein was incubated with the whole worm lysates of LD1(*ldls7*) and glutathione Sepharose 4B beads. After extensive washes, bound proteins were analyzed by western blotting with an anti-GFP antibody. Twenty percent of proteins used for the pull down reaction were shown as input.

### Immunoprecipitation (IP) and western blotting

To prepare *C. elegans* proteins, synchronized young adult worms were grown on a 9.5 cm plates at 20 °C, and washed off from the plates with M9 buffer. The worms were lysed by sonication in lysis buffer (50mMTris-HCl [pH 7.5], 150 mM NaCl, 1 mM EDTA, 0.5% NP-40, phosphatase inhibitors, and protease inhibitors) and then immunoprecipitated with anti-GFP antibody, followed by western blotting. For IP assay, total protein lysates were incubated overnight with corresponding antibodies with gentle shacking at 4 °C., followed by addition of 40 μl of Pure Proteome protein A/G Mix Magnetic Beads (Millipore) for another 3 h. The beads were resuspended in 60 μl of 2 × loading buffer and boiled for 10 min. Then the supernatant was subjected to SDS-PAGE, transferred to polyvinylidene fluoride membrane (Millipore) and visualized by using appropriate primary antibodies coupled with HRP-conjugated secondary antibodies by ECL reagent (GE Healthcare).

### *O-*GlcNAcylation assay and mass spectrometry (MS) analysis

For *O-*GlcNAcylation assay, the pGEX-6p-1plamids cloned with the wild type *skn-1* cDNA or each point mutants of *skn-1* cDNA, and the vectors pET28a(+) cloned with *C. elegans ogt-1* cDNA, were co-transfected into *E. coli* BL21(DE3) competent cells and selected on LB-agar plates containing 50 mg/l kanamycin and 100 mg/l ampicillin (Amresco). One colony was picked, and the protein expression was induced at 16 °C for 20 h with 0.1 mM isopropyl-β-D-thiogalactopyranoside (IPTG) after the absorbance at 600 nm reached 0.8. GST-tagged protein was purified using glutathione Sepharose 4B beads (GE Healthcare) and concentrated in a buffer containing 50 mM Tris-HCl (pH 7.6), 150 mM NaCl, 1% NP-40 and 10% glycerol. Purified proteins that were co-expressed with or without OGT-1 were subjected to western blotting analysis by using anti-*O-*GlcNAc antibody (abcam).

For mass spectrometry (MS) analysis, the Coomassie blue stained band of purified GST-SKN-1 co-expressed with HIS-OGT in BL21 (DE3) bacteria. was excised for LC-MS analysis performed in the company of Shanghai Applied Protein Technology.

### In *vitro* kinase assay

The peptides were incubated with GST-GSK-3 in buffer containing 20 mM Tris (pH 7.5), 5 mM DTT, 20 mM MgCl2, cold 200 μM ATP at 30 °C for 1 hour. The reaction was terminated by addition of SDS-Loading buffer and analyzed by western blotting.

### Statistical analysis

Statistical analysis was performed using GraphPad Prism 5 software (GraphPad Software, La Jolla, CA, USA). For the RT-qPCR assays, *p* values were determined by Student’s *t*-test. For the lifespan and oxidative stress resistance assays, *p* values were determined by log-rank test. For the nuclear localization of SKN-1B/C::GFP, a chi2 test was used, and the differences were considered as significance at *P* < 0.05.

## Additional Information

**How to cite this article:** Li, H. *et al*. *O*-GlcNAcylation of SKN-1 modulates the lifespan and oxidative stress resistance in *Caenorhabditis elegans. Sci. Rep.* **7**, 43601; doi: 10.1038/srep43601 (2017).

**Publisher's note:** Springer Nature remains neutral with regard to jurisdictional claims in published maps and institutional affiliations.

## Supplementary Material

Supplementary Information
