# Supplementary material for: O-GlcNAcylation of SKN-1 modulates the lifespan and oxidative stress resistance in Caenorhabditis elegans
Source: Sci Rep. 2017 Mar 8;7:43601. doi: 10.1038/srep43601 (PMC5341102; doi:10.1038/srep43601)
Supplement: Supplementary Information [file srep43601-s1.pdf]

**Supplementary information**

# *O*-GlcNAcylation of SKN-1 modulates the lifespan and oxidative stress resistance in *Caenorhabditis elegans*

Hongyuan Li[1], Xin Liu[2], Dan Wang[1], Liangping Su[1], Tingting Zhao[1], Zhongwei Li[2], Cong Lin[2], Yu Zhang[2], Baiqu Huang[2], Jun Lu[2*], Xiaoxue Li[1*]

[1] The Key Laboratory of Molecular Epigenetics of the Ministry of Education, Northeast Normal University, Changchun 130021, China;
[2] The Institute of Genetics and Cytology, Northeast Normal University, Changchun 130024, China;

[*]Corresponding author, lixx956@nenu.edu.cn; luj809@nenu.edu.cn.

**Supplementary Fig. S1 The changes of global *O*-GlcNAcylation level in the *SKN-1B/C::GFP* transgenic worms on *ogt-1(ok1474)* and *oga-1(ok1207)* background.**

The whole worm extracts of the SKN-1B/C::GFP transgenic worms on wild-type ($N_2$), *ogt-1(ok1474)* and *oga-1(ok1207)* background, were immunoblotted with anti-*O*-GlcNAc antibody (RL2). Actin protein was used as an internal reference.

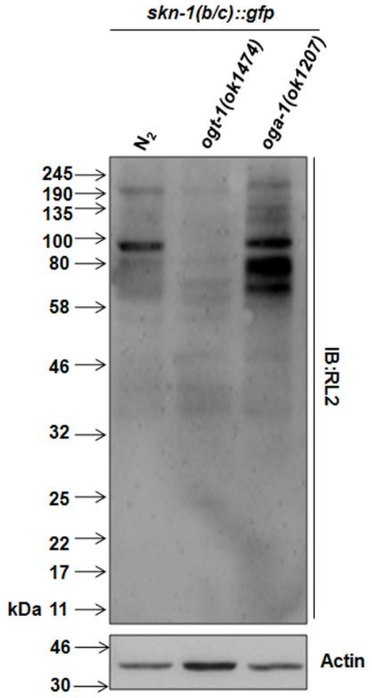


**Supplementary Fig. S2 The ROS level in the wild-type ($N_2$) was increased by 5 mM tBHP treatment.**

(**a**) The representative images of reactive oxygen species (ROS) formation induced by 5 mM tBHP treatment in wild-type ($N_2$). $N_2$ worms were exposed to M9 buffer or 5 mM tBHP for 90 minutes. Then, the level of ROS was assessed by incubation with 50 μM $H_2$DCF-DA at 20$^{o}$C for 30 min. (**b**) 5 mM tBHP treatment increased the ROS level in $N_2$. The fluorescence intensity was analyzed by Image J. The fluorescence intensity of N2 without tBHP treatment was utilized as control. **, $p < 0.01$. n, the number of worms for fluorescence intensity analysis.

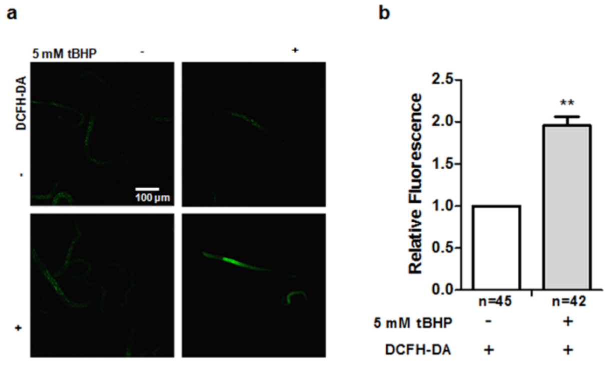

**Supplementary Fig. S3 The knockdown efficiency of *skn-1* RNAi in *oga-1(ok1207)*.**

*oga-1(ok1207)* worms were treated with *control* RNAi or *skn-1* RNAi, respectively. Then, the mRNA level of *skn-1* was measured by RT-qPCR. *act-1* was used as an internal reference. The representative data was from three independent experiments. ***, $p < 0.001$. *control* , the empty vector.

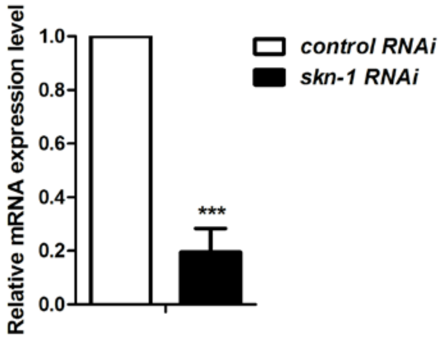


**Supplementary Fig. S4 Thr445, Ser446 and Ser449 of SKN-1 were the residues *O*-GlcNAcylated by OGT-1.**

**(a-b)** The reisdues of Thr445, Ser446, Ser449 were identified as the *O*-GlcNAcylation sites of SKN-1 by the mass spectrometry analysis from the fragmentation of the SKN-1 peptides 440aa-QQQTQTSPGSVTVTATATQSLFDPYHSQR-468aa.

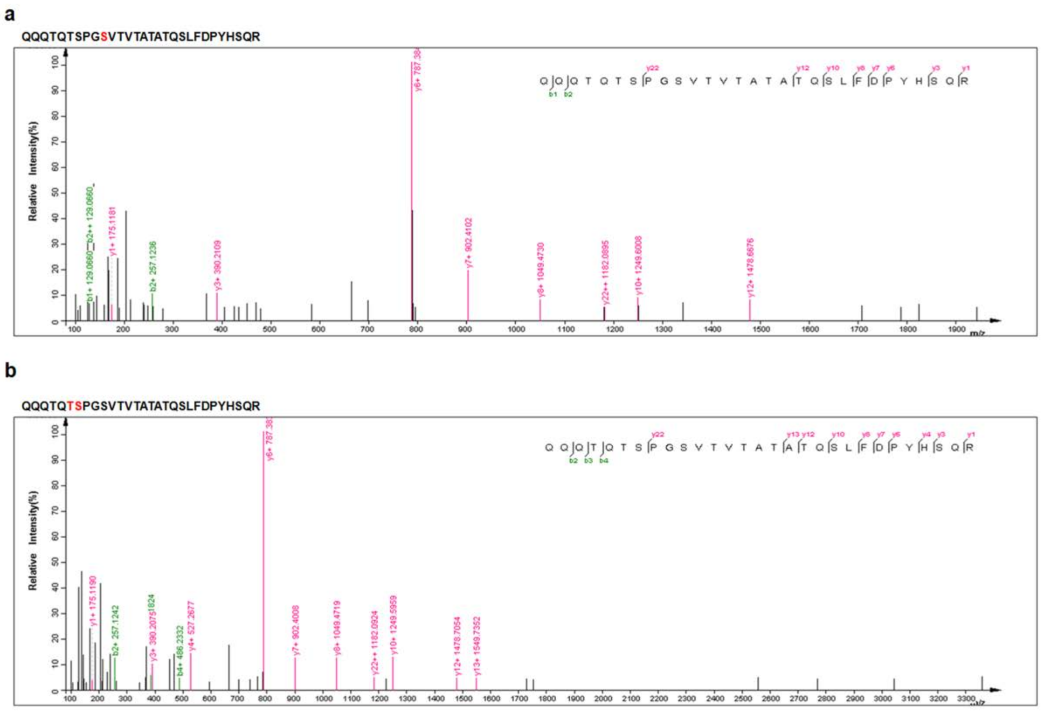


**Supplementary Fig. S5 The polential site subjected to phosphorylation within the sequence context, in which Ser470 and Thr493 are located.**

Ser483 was chosen to examine the phosphoryation level in response to *O*-GlcNAcylation of Ser470

and Thr493.

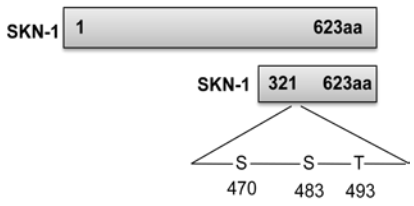


**Supplementary Fig. S6 The specificity and titire of the anti-phospho-SKN-1(Ser483) antibody recognizing phosphorylated Ser483 of SKN-1.**

The specificity and affinity of the antibody (Ser483 Pho) recognizing the phosphorylated Ser483 of SKN-1 was analyzed by ELISA analysis **(a)** and dot blot analysis **(b)**.The affinity-purified antibodies raised against the synthetic phosphorylated peptide (Pho-Peptide) containing 21aa of SKN-1. The peptide sequence: TTDSSSTCS(#)RLSSESPRYTSE. #, means the phosphorylated residue of Ser483 on SKN-1 protein. The synthetic non-phosphorylated peptide (Non-Pho-Peptide) was as the negative control. Pho-Peptide and Non-Pho-Peptide had the same amino acid sequence.

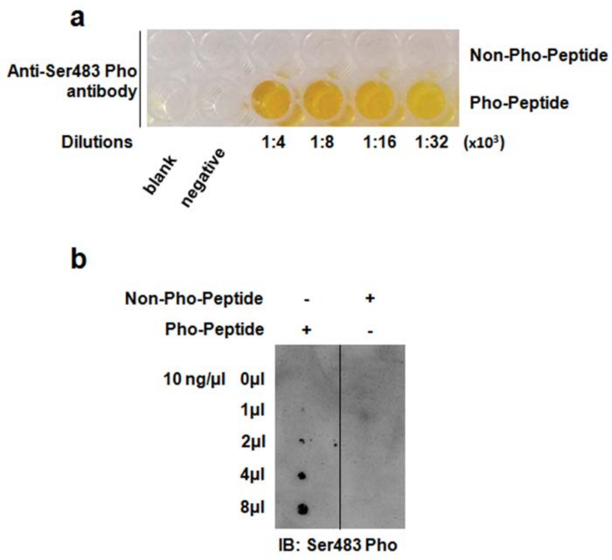


**Supplementary Fig. S7 Loss of *oga-1* decreased the phosphorylation level of SKN-1 at S483.**

The phosphorylation level of SKN-1 at Ser483 was detected by immunoprecipitation of SKN-1 from the *SKN-1B/C::GFP* worms and *oga-1(ok1207);SKN-1B/C::GFP* worms with anti-GFP antibody, followed by immunoblotting (IB) with anti-phospho-SKN-1(Ser483) antibody (Ser483 Pho) and anti-*O*-GlcNAc antibody.

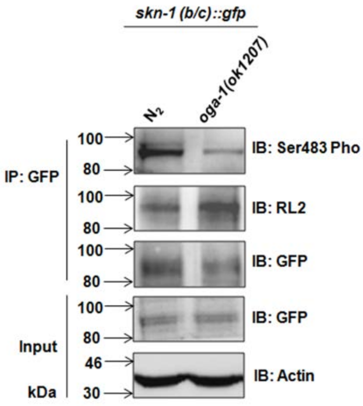


**Supplementary Fig. S8 Nrf2 was *O*-GlcNAcylated by human OGT.**

The HIS-tagged human OGT and GST-tagged Nrf2 were co-transformed in *Escherichia coli* BL21. The *O*-GlcNAc modification of Nrf2 was confirmed by immunoblotting (IB) with the anti-*O*-GlcNAc antibody.

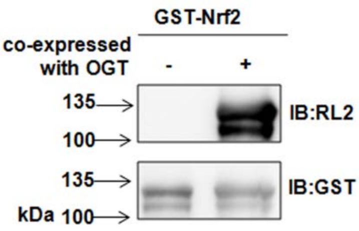


**Supplementary Fig. S9 Loss of *ogt-1* or *oga-1* did not change SKN-1 expression in the ASI neurons.**

The expression level of SKN-1 in the ASI neurons was not altered in *ogt-1(ok1474)* or *oga-1(ok1207)* worms, compared with the wild type worms. Red arrows denoted the position of the ASI neurons.

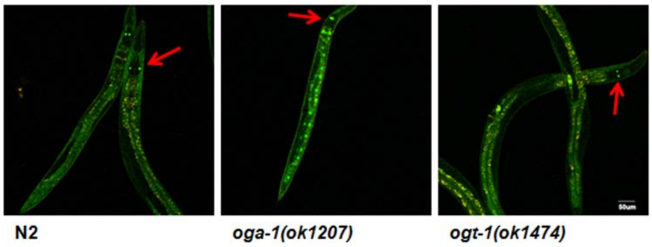


**Supplementary Fig. S10 The anti-OGT and anti-GFP antibodies reacted specifically with the proteins of OGT-1 and GFP-SKN-1 expressed in *C. elegans*.**

**(a)**The specificity of anti-OGT antibody recognizing *C. elegans* OGT-1 was confirmed by immunoblotting with the whole extract of the wild type worms. The arrow showed the band size of the *C.elegans* OGT-1. **(b)** The affinity specificity of anti-GFP antibody bound to *C. elegans* GFP-SKN-1 was verified by immunoblotting (IB) with the whole worm extract from *SKN-1B/C::GFP* transgenic

worms. The arrow showed the band size of the *C. elegans* GFP-SKN-1. Actin protein was used as an internal reference.

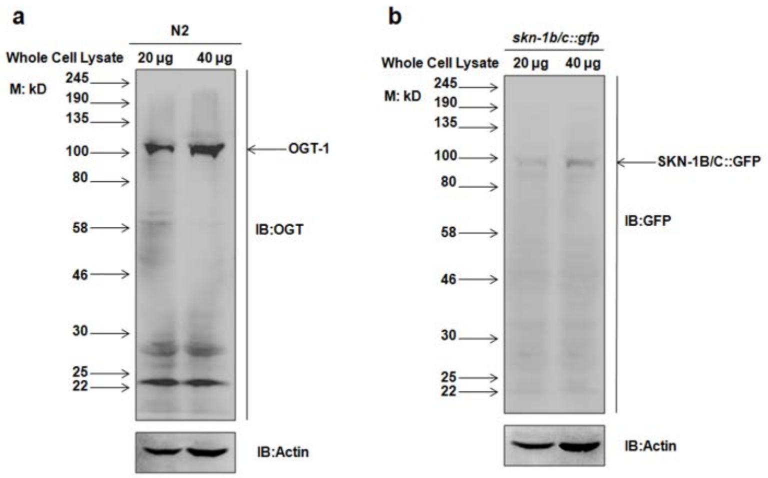

**Supplementary Tables. S1 *O*-GlcNAcylation of SKN-1 contributed to the extension of lifespan in *C. elegans*.**

| *O*-GlcNAcylation of SKN-1 contributed to the extension of lifespan in *C.elegans*. | | | | |
|---|---|---|---|---|
| Trial #1 | | | | |
| strains | Mean lifespan 20°C(days) | Median lifespan | *p* Value | Total Animals Died/Total |
| *N2[rol-6]* | 15.5 | 15 | — | 73/80 |
| *N2[SKN-1B/C S470A/T493A::GFP;rol-6]-1* | 15.3 | 15 | ns[a] | 71/75 |
| *N2[SKN-1B/C S470A/T493A::GFP;rol-6]-2* | 15.0 | 14.5 | ns[a] | 48/50 |
| *N2[SKN-1B/C::GFP;rol-6]-1* | 19.8 | 19 | < .0001[a] | 55/60 |
| *N2[SKN-1B/C::GFP;rol-6]-2* | 18.1 | 19 | < .0001[a] | 40/50 |
| *skn-1(zu67)[rol-6]* | 13.1 | 14 | — | 53/55 |
| *skn-1(zu67)[SKN-1B/C S470A/T493A::GFP;rol-6]-1* | 13.0 | 13 | ns[b] | 63/65 |
| *skn-1(zu67)[SKN-1B/C S470A/T493A::GFP;rol-6]-2* | 13.4 | 13 | ns[b] | 60/65 |
| *skn-1(zu67)[SKN-1B/C::GFP;rol-6]-1* | 18.7 | 19 | < .0001[b] | 68/70 |
| *skn-1(zu67)[SKN-1B/C::GFP;rol-6]-2* | 18.7 | 18 | < .0001[b] | 53/60 |
| Trial #2 | | | | |
| *N2[rol-6]* | 14.8 | 14.5 | — | 57/60 |
| *N2[SKN-1B/C S470A/T493A::GFP;rol-6]-1* | 15.5 | 15 | ns[a] | 41/50 |
| *N2[SKN-1B/C S470A/T493A::GFP;rol-6]-2* | 15.8 | 15 | ns[a] | 43/50 |
| *N2[SKN-1B/C::GFP;rol-6]-1* | 19.3 | 19 | < .0001[a] | 55/60 |
| *N2[SKN-1B/C::GFP;rol-6]-2* | 18.4 | 18 | < .0001[a] | 40/50 |
| *skn-1(zu67)[rol-6]* | 13.2 | 13 | — | 36/40 |
| *skn-1(zu67)[SKN-1B/C S470A/T493A::GFP;rol-6]-1* | 13.2 | 13 | ns[b] | 43/50 |
| *skn-1(zu67)[SKN-1B/C S470A/T493A::GFP;rol-6]-2* | 12.7 | 13 | ns[b] | 53/55 |
| *skn-1(zu67)[SKN-1B/C::GFP;rol-6]-1* | 18.6 | 19 | < .0001[b] | 55/60 |
| *skn-1(zu67)[SKN-1B/C::GFP;rol-6]-2* | 18.2 | 18 | < .0001[b] | 58/60 |
| Trial #3 | | | | |
| *N2[rol-6]* | 15.2 | 15 | — | 48/50 |
| *N2[SKN-1B/C S470A/T493A::GFP;rol-6]-1* | 15.1 | 15 | ns[a] | 55/60 |
| *N2[SKN-1B/C S470A/T493A::GFP;rol-6]-2* | 15.2 | 15 | ns[a] | 45/50 |
| *N2[SKN-1B/C::GFP;rol-6]-1* | 19.3 | 19 | < .0001[a] | 51/55 |
| *N2[SKN-1B/C::GFP;rol-6]-2* | 18.4 | 19 | < .0001[a] | 46/50 |
| *skn-1(zu67)[rol-6]* | 13.6 | 13 | — | 47/50 |
| *skn-1(zu67)[SKN-1B/C S470A/T493A::GFP;rol-6]-1* | 13.7 | 14 | ns[b] | 56/60 |
| *skn-1(zu67)[SKN-1B/C S470A/T493A::GFP;rol-6]-2* | 13.2 | 13 | ns[b] | 43/50 |
| *skn-1(zu67)[SKN-1B/C::GFP;rol-6]-1* | 18.8 | 19 | < .0001[b] | 57/60 |
| *skn-1(zu67)[SKN-1B/C::GFP;rol-6]-2* | 18.3 | 18 | < .0001[b] | 57/60 |

| Total | | |
|---|---|---|
| strains | Mean lifespan ± SEM 20°C (days) | *p* Value |
| *N2[rol-6]* | 15.1 ± 0.2 | — |
| *N2[SKN-1B/C S470A/T493A::GFP;rol-6]-1* | 15.2 ± 0.1 | ns[a] |
| *N2[SKN-1B/C S470A/T493A::GFP;rol-6]-2* | 15.3 ± 0.2 | ns[a] |
| *N2[SKN-1B/C::GFP;rol-6]-1* | 19.4 ± 0.1 | < .0001[a] |
| *N2[SKN-1B/C::GFP;rol-6]-2* | 18.3 ± 0.1 | < .0001[a] |
| *skn-1(zu67)[rol-6]* | 13.3 ± 0.1 | — |
| *skn-1(zu67)[SKN-1B/C S470A/T493A::GFP;rol-6]-1* | 13.3 ± 0.2 | ns[b] |
| *skn-1(zu67)[SKN-1B/C S470A/T493A::GFP;rol-6]-2* | 13.1 ± 0.1 | ns[b] |
| *skn-1(zu67)[SKN-1B/C::GFP;rol-6]-1* | 18.7 ± 0.1 | < .0001[b] |
| *skn-1(zu67)[SKN-1B/C::GFP;rol-6]-2* | 18.4 ± 0.1 | < .0001[b] |

Corresponds to the data in Figures 4d, 4e. SEM: standard error of the mean. Median life span refers to the day at which 50% of the population is dead. The total number of observations equals the number of animals that died plus the number censored. Animals that crawled off the plate, bagged, or burst were censored and were therefore excluded from all analysis. *p* values were calculated as follows: [a]*N2[rol-6]*, [b]*skn-1(zu67)[rol-6]*. All statistical analysis was carried out using Graphpad Prism 5.0a software. The logrank (Mantel-Cox) test was used for statistical analysis.

## Supplementary **Table. S2 *O*-GlcNAcylation of SKN-1 promoted the oxidative stress resistance in *C. elegans*.**

| *O*-GlcNAcylation of SKN-1 promoted the oxidative stress resistance in *C.elegans*. | | | |
|---|---|---|---|
| Trial #1 | | | |
| strains | Mean lifespan 20℃(hours) | *p* Value | Total Animals Died/Total |
| *$N_2$[rol-6]* | 8.8 | — | 37/40 |
| *$N_2$[SKN-1B/C S470A/T493A::GFP;rol-6]-1* | 8.7 | ns[a] | 51/53 |
| *$N_2$[SKN-1B/C S470A/T493A::GFP;rol-6]-2* | 8.4 | ns[a] | 67/70 |
| *$N_2$[SKN-1B/C::GFP;rol-6]-1* | 10.1 | < .0001[a] | 41/45 |
| *$N_2$[SKN-1B/C::GFP;rol-6]-2* | 10.3 | < .0001[a] | 59/60 |
| *skn-1(zu67)[rol-6]* | 6.9 | — | 53/55 |
| *skn-1(zu67)[SKN-1B/CS470A/T493A::GFP;rol-6]-1* | 6.7 | ns[b] | 56/60 |
| *skn-1(zu67)[SKN-1B/CS470A/T493A::GFP;rol-6]-2* | 6.6 | ns[b] | 43/49 |
| *skn-1(zu67)[SKN-1B/C::GFP;rol-6]-1* | 8.9 | < .0001[b] | 47/49 |
| *skn-1(zu67)[SKN-1B/C::GFP;rol-6]-2* | 9.0 | < .0001[b] | 37/40 |
| Trial #2 | | | |
| *$N_2$[rol-6]* | 8.6 | — | 36/40 |

| | | | |
|---|---|---|---|
| $N_2$*[SKN-1B/C S470A/T493A::GFP;rol-6]-1* | 8.6 | ns[a] | 51/55 |
| $N_2$*[SKN-1B/C S470A/T493A::GFP;rol-6]-2* | 8.7 | ns[a] | 56/60 |
| $N_2$*[SKN-1B/C::GFP;rol-6]-1* | 10.5 | < .0001[a] | 38/40 |
| $N_2$*[SKN-1B/C::GFP;rol-6]-2* | 11.0 | < .0001[a] | 39/40 |
| *skn-1(zu67)[rol-6]* | 6.7 | — | 43/50 |
| *skn-1(zu67)[SKN-1B/CS470A/T493A::GFP;rol-6]-1* | 6.8 | ns[b] | 38/40 |
| *skn-1(zu67)[SKN-1B/CS470A/T493A::GFP;rol-6]-2* | 7.0 | ns[b] | 46/50 |
| *skn-1(zu67)[SKN-1B/C::GFP;rol-6]-1* | 8.6 | < .0001[b] | 46/50 |
| *skn-1(zu67)[SKN-1B/C::GFP;rol-6]-2* | 8.8 | < .0001[b] | 33/40 |
| Trial #3 | | | |
| $N_2$*[rol-6]* | 9.0 | — | 68/70 |
| $N_2$*[SKN-1B/C S470A/T493A::GFP;rol-6]-1* | 8.8 | ns[a] | 65/68 |
| $N_2$*[SKN-1B/C S470A/T493A::GFP;rol-6]-2* | 8.4 | ns[a] | 50/55 |
| $N_2$*[SKN-1B/C::GFP;rol-6]-1* | 10.2 | < .0001[a] | 41/45 |
| $N_2$*[SKN-1B/C::GFP;rol-6]-2* | 10.4 | < .0001[a] | 40/45 |
| *skn-1(zu67)[rol-6]* | 6.6 | — | 33/35 |
| *skn-1(zu67)[SKN-1B/CS470A/T493A::GFP;rol-6]-1* | 6.7 | ns[b] | 52/55 |
| *skn-1(zu67)[SKN-1B/CS470A/T493A::GFP;rol-6]-2* | 6.4 | ns[b] | 42/45 |
| *skn-1(zu67)[SKN-1B/C::GFP;rol-6]-1* | 8.7 | < .0001[b] | 57/60 |
| *skn-1(zu67)[SKN-1B/C::GFP;rol-6]-2* | 9.3 | < .0001[b] | 36/40 |

Total

| strains | Mean lifespan ± SEM 20$^{o}$C(days) | *p* Value |
|---|---|---|
| $N_2$*[rol-6]* | 8.8 ± 0.1 | — |
| $N_2$*[SKN-1B/C S470A/T493A::GFP;rol-6]-1* | 8.7 ± 0.1 | ns[a] |
| $N_2$*[SKN-1B/C S470A/T493A::GFP;rol-6]-2* | 8.5 ± 0.1 | ns[a] |
| $N_2$*[SKN-1B/C::GFP;rol-6]-1* | 10.2 ± 0.1 | < .0001[a] |
| $N_2$*[SKN-1B/C::GFP;rol-6]-2* | 10.5 ± 0.2 | < .0001[a] |
| *skn-1(zu67)[rol-6]* | 6.7 ± 0.1 | — |
| *skn-1(zu67)[SKN-1B/CS470A/T493A::GFP;rol-6]-1* | 6.7 ± 0.1 | ns[b] |
| *skn-1(zu67)[SKN-1B/CS470A/T493A::GFP;rol-6]-2* | 6.6 ± 0.2 | ns[b] |
| *skn-1(zu67)[SKN-1B/C::GFP;rol-6]-1* | 8.7 ± 0.1 | < .0001[b] |
| *skn-1(zu67)[SKN-1B/C::GFP;rol-6]-2* | 9.0 ± 0.1 | < .0001[b] |

Corresponds to the data in Figures 4f, 4g. SEM: standard error of the mean. The total number of observations equals the number of animals that died plus the number censored. Animals that crawled off the plate, bagged, or burst were censored and were therefore excluded from all analysis. *p* values were calculated as follows: [a]*N2[rol-6]*, [b]*skn-1 (zu67)[rol-6]*. All statistical analysis was carried out using Graphpad Prism 5.0a software. The logrank (Mantel-Cox) test was used for statistical analysis.

## Supplementary Methods

### RNAi

RNAi clones were grown in LB with 12.5 μg/ml tetracycline and 100 μg/ml ampicillin. On the next day, cultures were diluted and grown to an OD600 of 1, and induced with 4 mM isopropyl-β-D-thiogalactopyranoside (IPTG). This culture was used to seed plates containing ampicillin, and additional IPTG, and then left to dry for 1-2 days at room temperature.

### Assessment of ROS levels

To detect the level of reactive oxygen species (ROS), $N_2$ worms were exposed to 5 mM tBHP or M9 buffer for 90min. Then, the worms were washed with M9 buffer for 3 times and soaked in 0.5 mL M9 buffer containing 50 μM 2, 7-Dichlorodihydrofluorescein ($H_2$DCF-DA, Sigma) for 30 min. The worms were then washed with M9 buffer for 3 times, and applied to take pictures by OLYMPUS confocal microscope. The fluorescence intensity was analyzed by Image J. The fluorescence intensity of N2 without tBHP treatment was utilized as control.

### The primer sequences for RT-qPCR

*gcs-1* (forward):5’-aatcgattcctttggagacc-3’
*gcs-1* (reverse): 5’-atgtttgcctcgacaatgtt-3’
*gst-4* (forward): 5’-cccattttacaagtcgatgg-3’
*gst-4*(reverse): 5’-cttcctctgcagtttttcca-3’
*gst-7* (forward):5’-aggacaacagaatcccaaagg-3’
*gst-7* (reverse): 5’-agcaaatcccatcttcaccat-3’
*act-1* (forward): 5’-tcggtatgggacagaaggac-3’
*act-1* (reverse): 5’-catcccagttggtgacgata-3’
